# Assessing reliability and validity of different stiffness measurement tools on a multi-layered phantom tissue model

**DOI:** 10.1038/s41598-023-27742-w

**Published:** 2023-01-16

**Authors:** Katja Bartsch, Andreas Brandl, Patrick Weber, Jan Wilke, Sabine F. Bensamoun, Wolfgang Bauermeister, Werner Klingler, Robert Schleip

**Affiliations:** 1 Department of Sport Science and Sport, Friedrich-Alexander Universität Erlangen-Nürnberg, Erlangen, Germany; 2 Conservative and Rehabilitative Orthopedics, Department of Sport and Health Sciences, Technical University of Munich, Munich, Germany; 3 Department of Sports Medicine, Faculty for Psychology and Human Movement Science, Institute for Human Movement Science, University of Hamburg, Hamburg, Germany; 4 Department for Medical Professions, Diploma Hochschule, Bad Sooden-Allendorf, Germany; 5 Osteopathic Research Institute, Osteopathie Schule Deutschland, Hamburg, Germany; 6 Department for Physical Activity in Public Health, Institute of Movement and Neurosciences, German Sport University Cologne, Cologne, Germany; 7 Department of Movement Sciences, University of Klagenfurt, Klagenfurt, Austria; 8 Institute of Occupational, Social and Environmental Medicine, Goethe University, Frankfurt, Frankfurt/Main, Germany; 9 Université de Technologie de Compiègne, CNRS UMR 7338, Biomechanics and Bioengineering Laboratory, Sorbonne University, Compiègne, France; 10 Charkiv National Medical University, Charkiv, Ukraine; 11 SRH Hospital, Sigmaringen, Germany; 12 Experimental Anaesthesiology, Ulm University, Ulm, Germany

**Keywords:** Preclinical research, Skeletal muscle

## Abstract

Changes in the mechanical properties (i.e., stiffness) of soft tissues have been linked to musculoskeletal disorders, pain conditions, and cancer biology, leading to a rising demand for diagnostic methods. Despite the general availability of different stiffness measurement tools, it is unclear as to which are best suited for different tissue types and the related measurement depths. The study aimed to compare different stiffness measurement tools’ (SMT) reliability on a multi-layered phantom tissue model (MPTM). A polyurethane MPTM simulated the four layers of the thoracolumbar region: cutis (CUT), subcutaneous connective tissue (SCT), fascia profunda (FPR), and erector spinae (ERS), with varying stiffness parameters. Evaluated stiffness measurement tools included Shore Durometer, Semi-Electronic Tissue Compliance Meter (STCM), IndentoPRO, MyotonPRO, and ultrasound imaging. Measurements were made by two independent, blinded examiners. Shore Durometer, STCM, IndentoPRO, and MyotonPRO reliably detected stiffness changes in three of the four MPTM layers, but not in the thin (1 mm thick) layer simulating FPR. With ultrasound imaging, only stiffness changes in layers thicker than 3 mm could be measured reliably. Significant correlations ranging from 0.70 to 0.98 (all *p* < 0.01) were found. The interrater reliability ranged from good to excellent (ICC(2,2) = 0.75–0.98). The results are encouraging for researchers and clinical practitioners as the investigated stiffness measurement tools are easy-to-use and comparatively affordable.

## Introduction

Low back pain is the leading cause of years lived with disability, burdening health care systems worldwide^1^. Historically, soft tissues in general and the connective tissues of the thoracolumbar region in particular have received little attention when attempting to clarify the pathophysiological mechanisms of this condition. In recent years, however, research has shed light on the layered soft tissue structures of the low back and their biomechanical characteristics as they contribute to low back health^2–6^.

Soft tissue stiffness is a mechanical property, which is defined as a material’s resistance to deformation^7^. Changes in the mechanical properties of soft tissues have been linked to musculoskeletal disorders, injuries, pain conditions, and cancer biology, leading to a rising demand in diagnostic methods for research and clinical practice^2,8–13^. However, little data exists to provide evidence-based recommendations for current stiffness measurement tools (SMT), requiring further research investigating their measurement properties^14,15^. Prior research has suggested to use material phantoms with known viscoelastic properties to obtain valuable results regarding the reliability and concurrent validity of SMT^16–18^.

The use of tissue SMT provides a comparatively cost-effective and easy-to-use option for research and clinical practice. Current technologies comprise methods of indentation, myotonometry, as well as ultrasound imaging^14,19^.

To our knowledge, none of these devices have been tested for reliability on a material phantom model representing the layered soft tissue structures of the human low back area. Analog to ‘The Princess of the Pea’ fairy tale (where the princess demonstrates her sensitivity to feel the slightest stiffness change created by a pea through layers of bedding and thereby proves herself to be a princess) it can be assumed that the sensitivity of a stiffness assessment may be altered when the tissue layer of interest is positioned underneath one or several other tissue layers. In our study, we aimed to create a multi-layered material phantom tissue model (MPTM) mimicking the different layers of the thoracolumbar region. The present study’s objective was to evaluate the reliability of various tissue stiffness measurement devices on the MPTM.

## Materials and methods

### Multi-layered material phantom tissue model

**Figure 1:**
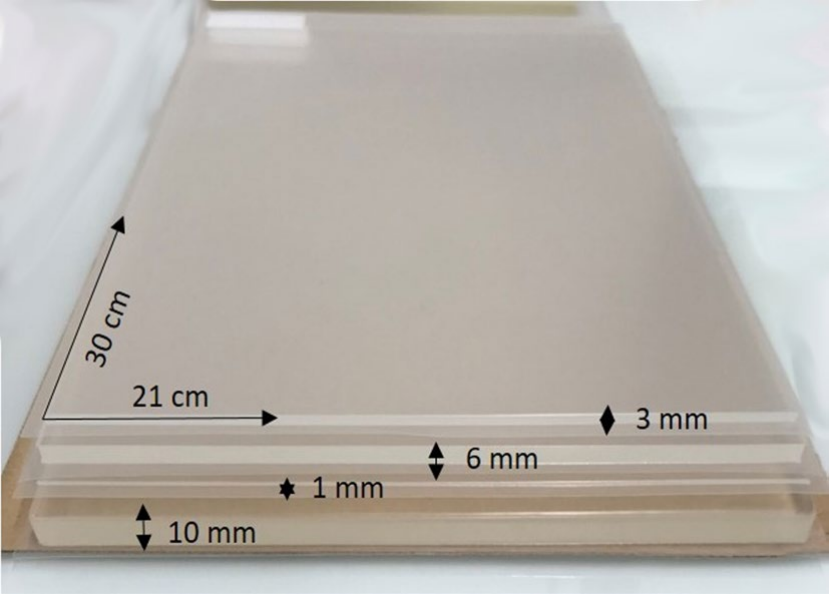
Multi-layered phantom tissue model.

**Figure 2:**
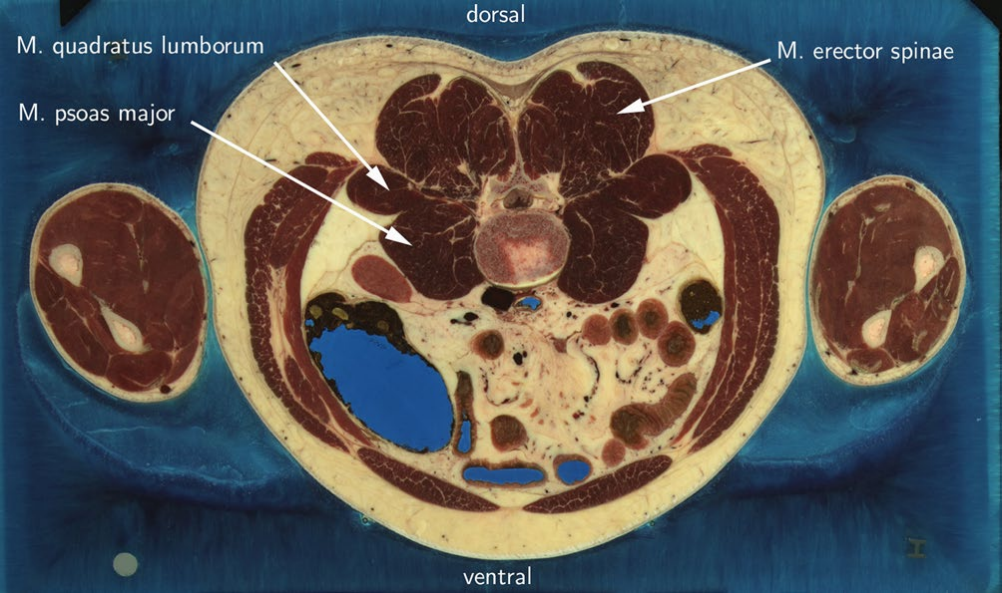
Cross-section of L3 region. Photo: Modified from Visible Human Project of U.S. National Library of Medicine^23^, accessed through NPAC/OLDA Visible Human Viewer^24^ with permission.

A multi-layered phantom model was developed to mimic the soft tissue layers of the human thoracolumbar region (Fig. 1). The model simulated four human tissue layers: cutis (CUT), subcutaneous connective tissue (SCT), fascia profunda (FPR), and erector spinae (ERS). A literature search yielded typical values for the thickness of those tissue layers located lateral to the spinous process of the L3 vertebra, as this measurement site has been used by prior research for stiffness evaluation^4,20–22^ (Fig. 2). Accordingly, a thickness of 3 mm, 6 mm, and 1 mm was chosen for CUT, SCT, and FPR respectively. For the ERS—as the most inferior of the four layers—a thickness of 10 mm was chosen, since preliminary explorations of our group had revealed that thickness as upper limit of a reliable assessment of the ultrasound assessment method. Typical stiffness values for the four layers were determined using literature searches as well. Gel pad layers with the identified typical thickness and stiffness values constituted the default measurement set. To mimic stiffness alterations, nine additional phantoms with varying stiffness parameters were produced for each tissue layer, resulting in 40 phantom layer variations overall. The parameters for the artificial stiffness changes for the four tissue layers were determined individually for each layer.

All gel pad phantoms measured 30 × 21 cm, were manufactured from polyurethane material and coated with a 25 µm thick polyurethane foil (Technogel Germany GmbH, Berlingrode). Stiffness values were specified in Shore OOO values, which describe very soft plastic and rubber materials. For further analysis, Shore OOO values were converted to Young’s modulus in kPasc^25^.

### Tissue stiffness measurement tools

#### Shore durometer

The Shore Durometer (Type 1600-OO, Rex Gauge, Brampton, ON, Canada) is a standard device for measuring the hardness of various non-metallic materials including rubber and plastic. The instrument contains a spring-loaded interior that senses hardness by applying an indentation load on the material through a probe tip (Ø 2.4 mm). For measurements, the durometer is held perpendicular to the medium and rested by gravity against the material. Hardness in degrees of Shore OO from 0 to 100 can be read from the analogue dial of the device, with lower Shore values indicating a softer material^26^.

#### Semi-electronic tissue compliance meter

The Semi-electronic Tissue Compliance Meter (STCM, Technical University of Chemnitz, Germany) consists of a force gauge (probe tip surface of 1 cm^2^) and a platform ring (Ø 8 cm), which slides downward on the force gauge shaft. Through a scale with millimeter increments equipped with a fixable ring, a desired penetration depth can be set. The device furthermore contains a button cell which is used to generate a beep signal upon contact between the ring and the disk. To perform a measurement, the probe tip is placed on the underlying tissue and the examiner applies a downward force on the top of the device until the disk and the ring are brought into contact. This contact generates a beep sound, indicating the end of the measurement. The applied force (in N) can be read from the analogue display of the force gauge. The known penetration force and the applied force can be used to analyze the force–deformation relationship of the material or tissue at hand^16,27^.

#### IndentoPRO

The IndentoPRO is a digital indentometer (Fascia Research Group, Ulm University; Department of Human Movement Sciences, University of Chemnitz, Germany). The device comprises a device body with a load cell (Compression Load Cell FX1901, TE Connectivity, Schaffhausen, Switzerland) and a membrane potentiometer (ThinPot 10kOhm, Spectra Symbol, Salt Lake City, USA) to measure the resistance force and displacement of a circular indentation probe (Ø 11.3 mm)^28,29^. To perform a measurement, an indentation depth is selected, and the probe is positioned on the material. The examiner then applies force on top of the device until a beep signal indicates the end of the measurement. Tissue stiffness is defined by the slope of the relationship between indentation depth and force increase. Stiffness values can be read from a digital display and are specified in N/mm, with lower values indicating lower tissue stiffness^28,30^.

#### MyotonPRO

The MyotonPRO is a digital palpation device (MyotonAS; Tallinn, Estonia), consisting of a device body and an indentation probe (Ø 3 mm). Through the probe, a pre-pressure (0.18 N) is applied to the surface that causes the material underneath to be compressed. A mechanical impulse (0.4 N, 15 ms) is then released by the device, deforming the medium for a short interval. The tissues respond back with a damped oscillation that is recorded by the accelerometer in the MyotonPRO device. Stiffness is recorded in N/m and can be read from a digital display. Lower values indicate lower tissue stiffness^18,31^.

#### Ultrasound with attached transducer

As described by Jafari and colleagues^32^ ultrasound images can be recorded in two states: with stress and without stress. For the stress state scenario, compressive stress is imposed by the ultrasound transducer (Philips Lumify with L12-4 linear transducer; contact area 19 × 43.5 mm). A force gauge attached through a ring holder measures the applied force (Digital Force Gauge FL-S-100, Kern & Sohn GmbH, Balingen, Germany). The ultrasound images with and without stress states are compared and the length of a vertical line perpendicular to the multi-layered phantom tissue model surface is measured. To determine strain, the measured length of the vertical lines with and without stress are used. Stress is calculated by dividing the applied force (in N) by the transducer contact area. Tissue stiffness is determined as elastic modulus, using the relation between stress and strain under the assumption that stiffness follows linear behavior.

### Measurements

Material phantoms were placed on top of each other according to their natural sequence. The default measurement set served as starting point. To mimic stiffness changes in the MPTM, 10 different layer variants representing 10 different stiffness parameters were exchanged one by one, with the other three layers remaining in default measurement set configuration (example: the 10 gel pads variants for CUT were exchanged and measured one by one with all measurement devices, while SCT, FPR and ERS remained in default measurement set configuration; analogous procedure was followed for the other layers). Measurements were performed by two investigators in a blinded manner, meaning that examiners were not aware of the stiffness parameters of each gel pad.

For the Shore Durometer, the average of five consecutive measurements was used for data analysis^33^. Taken measurements were converted to Young’s modulus in kPasc^25^. For the semi-electronic STCM, three consecutive measurements at 15 mm penetration depth were taken and averaged for data analysis^16^. For the IndentoPRO, three consecutive measurements were taken^34^ at four different indentation depths (2 mm, 5 mm, 8 mm, 10 mm) and averaged for analysis. Measurements were only accepted when the coefficient of variation (CV) had maximum value of 5%. Investigators trained beforehand to conduce indentations with a consistent force rate of 10 N/s to achieve said level of CV. For the MyotonPRO, the average value of five consecutive measurements was determined^15,18^. For the ultrasound, measurements with 0 kPa, 5 kPa, 10 kPa, and 15 kPa pressure were taken^32^.

### Data analysis and statistics

All descriptive data are means ± standard deviation (SD). To investigate the relationship between relative changes of stiffness determined with the SMTs and artificial relative Young’s modulus changes of the MPTM, Pearson’s product-moment correlation coefficients were calculated for normal distributed data and Spearman’s rank correlation for data that violated normal distribution assumptions. Linear regression analysis (with log 10 transformation for non-normally distributed data) was performed with MPTM measurement as dependent variable and respective SMTs measurement as independent variable. According to Cohen^35^, the resulting values were interpreted as ‘small ‘ (0.1 to 0.3), ‘medium’ (0.3 to 0.5) or ‘large’ (0.5 to 1.0) correlations. Intraclass correlation coefficient (ICC) estimates between MPTM and IndentoPRO measurement and their 95% CI were calculated using the R package “irr” version 0.84.1 based on a 2-way mixed-effects model with absolute agreement. Non-normally distributed data were log-10-transformed. Resulting ICC values were interpreted according to Fleiss^36^ as ‘poor’ (< 0.4), ‘fair to good’ (0.4 to 0.75) and ‘excellent’ (> 0.75). For relative reliability^37^, the corresponding standard errors of measurement (SEM) were estimated using the formula:

1 $$SEM = SD * \sqrt {\left( {1 - ICC} \right)} .$$

The MDC was estimated by reference to the SEM using the formula (2)^38^:

2 $$MDC = 1.96*\sqrt 2 *SEM = 1.96*SD*\sqrt {\left( {1 - ICC} \right)} .$$

To complement the ICC reliability data, the Bland and Altman test with limits of agreement was conducted between the two raters and graphed.

An alpha level of 0.05 was used to determine statistical significance. LibreOffice Calc version 6.4.7.2 (Mozilla Public License v2.0) was used for descriptive statistics. Inferential statistics were carried out with the software R, version 3.4.1 (R Foundation for Statistical Computing, Vienna, Austria).

## Results

**Figure 3:**
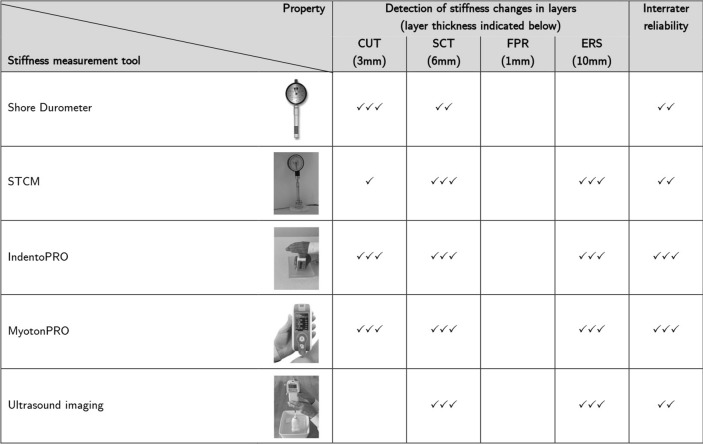
CUT cutis. SCT subcutaneous connective tissue. FPR fascia profunda. ERS erector spinae. Blank denotes “no reliable measurement possible”. ✓ denotes moderate correlation (> 0.4). ✓✓ denotes strong. correlation (> 0.7). ✓✓✓ denotes very strong correlation (> 0.9). Photos (1st to 5th row): rows 1–3: fasciaresearch.org with permission; myotonpro.com with permission, fasciareasearch.org with permission.

**Figure 4:**
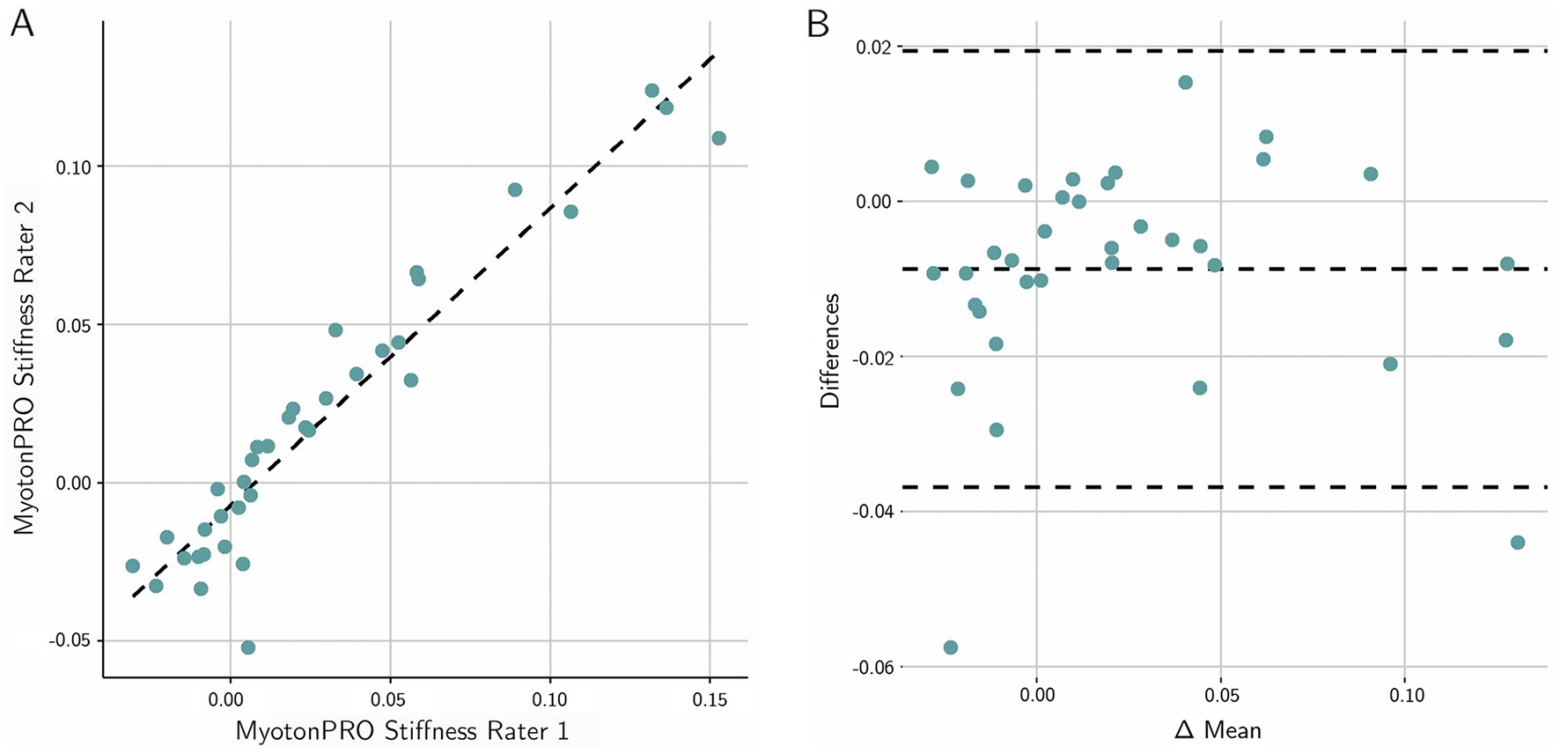
(**A**) Scatter plot of the agreement between the two raters for the MyotonPRO. (**B**) Bland-Altmann plot of the mean differences between the raters. The dashed line in the middle represents the mean difference; the lines above and below show the 95% limits of agreement. The values indicate the relative stiffness changes.

**Figure 5:**
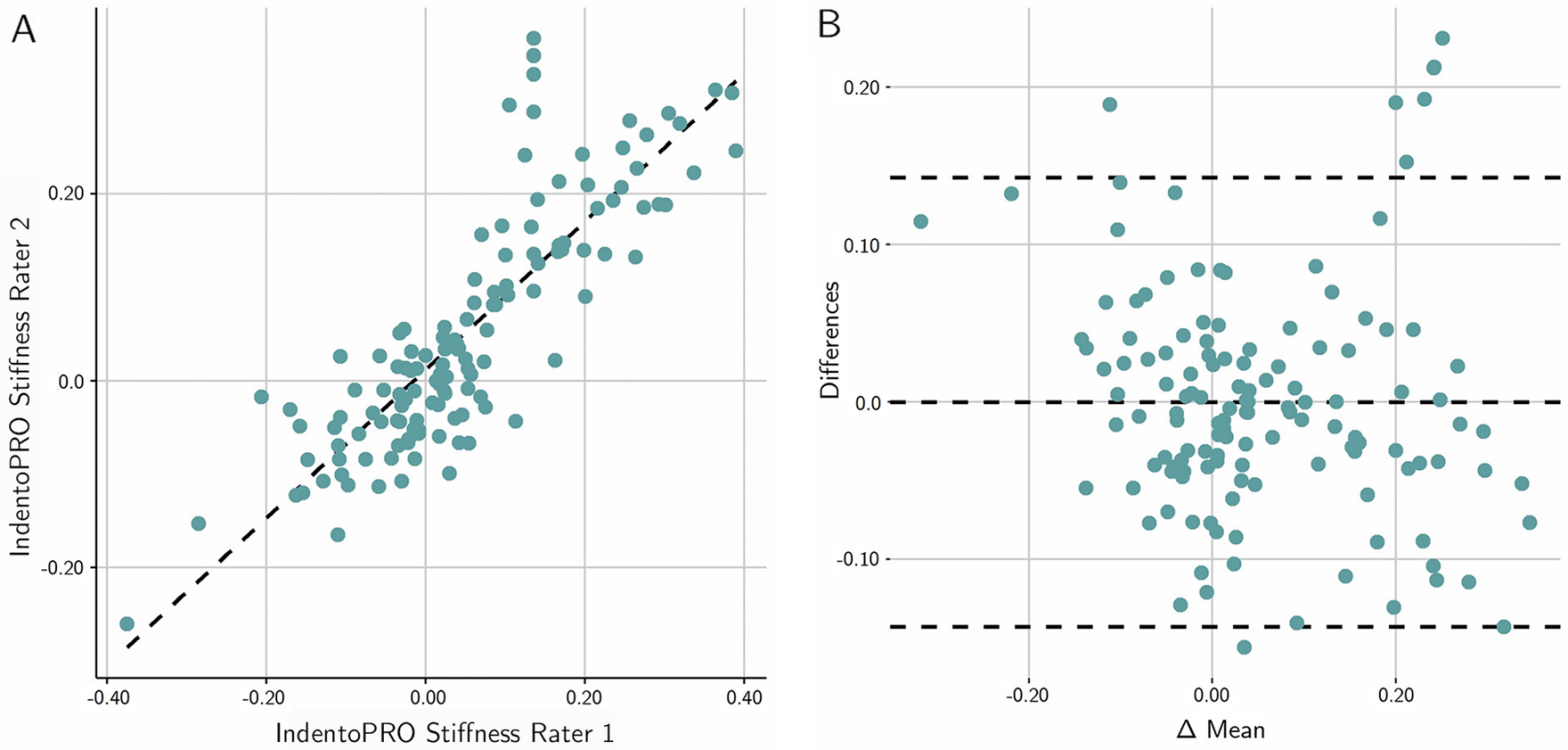
(**A**) Scatter plot of the agreement between the two raters for the IndentoPRO. (**B**) Bland-Altmann plot of the mean differences between the raters. Legend: see Fig. 4.

Significant correlations with artificial Young’s modulus changes of the MPTM were found for stiffness changes in all layers of the MPTM except for the FPR layer, with effect sizes ranging from 0.70 to 0.98 (all *p* < 0.01). The interrater reliability for all layers except FPR was good to excellent (ICC (2,2) = 0.75–0.98). Figure 3 gives an overview of the results. Figure 4 graphs results for the Bland and Altman test for MyotonPRO, Fig. 5 depicts Bland and Altman test results for IndentoPRO.

The following values relate to CUT, SCT, and ERS layers.

Correlations for the Shore Durometer ranged from 0.53 to 0.92 (0.92 for detecting artificial stiffness changes in CUT, 0.77 for detecting changes in SCT, 0.53 for detecting changes in ERS). Interrater reliability (ICC) was good and ranged from 0.51 (ERS) to 0.69 (CUT).

For STCM, correlations ranged from 0.05 to 0.93. Measurements taken with 10 mm penetration depth showed higher correlations (0.70 for changes in CUT, 0.93 for SCT layer, 0.92 for ERS) than measurements with 5 mm penetration depth (0.55, 0.65, and 0.05 respectively). Excellent interrater-variability values were obtained for ERS (0.93), and SCT (0.8) (both related to10 mm penetration depth).

MyotonPRO stiffness (N/m) showed the highest correlations throughout all devices, amounting to 0.94 for changes in CUT, 0.98 for SCT and 0.91 for ERS. Interrater reliability for MyotonPRO was excellent throughout all MPTM layers (0.98 for CUT, 0.99 for SCT, 0.94 for ERS).

Correlations for IndentoPRO were also large. Highest correlations were found for measurements with a penetration depth of 10 mm (0.90 for stiffness changes in CUT, 0.98 for SCT, 0.97 for ERS). Values for 2 mm penetration depth amounted to 0.88 (cutis), 0.97 (SCT), 0.96 (ERS). Values for 5 mm penetration depth were 0.71 (CUT), 0.97 (SCT), 0.96 (ERS). Correlations for 8 mm penetration depth were calculated at 0.87 (CUT), 0.98 (SCT), and 0.92 (ERS). Interrater reliability was excellent for all penetration depths except CUT at 5 mm penetration depth (ICC = 0.51) and SCT at 10 mm penetration depth (ICC = 0.71).

Ultrasound with attached force gauge showed large correlations for SCT (0.80–0.95, for varying pressure levels), and ERS (0.87–0.96). Interrater reliability was excellent for ERS (all applied pressure parameters) and SCT (at 15 kPasc). Interrater reliability for SCT was good for 5 kPasc and 10 kPasc pressures.

The detailed results are presented in Table 1.

**Table 1:** **Results.**

SMT	Depth/Pressure	Layer	SMT reliability				Interrater reliability			
			Cor	Linear regression formula	R^2^	*p* value	ICC	95% CI	SEM	MDC
Shore Durometer	**NA**	**1**	0.92***	(− 0.0525 + 0.349 * RV)	**0.85**	** < 0.001**	**0.69**	**(− 0.42 to 0.93)**	**0.16**	**0.45**
STCM	5 mm	1	0.55 +	(0.1211 + 0.176 * RV)	0.35	0.096	0.12 +	(− 0.18 to 0.59)	0.42	1.15
	10 mm	1	0.70* +	(0.0228 + 0.0758 * RV)	0.48	0.037	0.18 +	(− 0.31 to 0.7)	0.10	0.28
MyotonPRO Stiffness	**NA**	**1**	0.94***	(0.0061 + 0.0757 * RV)	**0.88**	** < 0.001**	0.98***	**(0.86 to 1)**	**0.01**	**0.02**
IndentoPRO	**2 mm**	**1**	0.88**	(− 0.063 + 0.1934 * RV)	**0.78**	**0.002**	0.78*	**(0.05 to 0.95)**	**0.09**	**0.24**
	5 mm	1	0.71*	(− 0.039 + 0.1867 * RV)	0.50	0.032	0.51	(− 0.61 to 0.88)	0.20	0.56
	**8 mm**	**1**	0.87**	(0.0176 + 0.0942 * RV)	**0.75**	**0.0025**	0.76*	**(− 0.11 to 0.95)**	**0.04**	**0.10**
	**10 mm**	**1**	0.90**	(− 0.0222 + 0.0956 * RV)	**0.81**	** < 0.001**	0.82*	**(0.24 to 0.96)**	**0.03**	**0.09**
Ultrasound	5 kPasc	1	NA	NA	NA	NA	NA	NA	NA	NA
	10 kPasc	1	0.20 +	(− 0.0158 + 0.0715 * RV)	0.01	0.803	0.08	(− 0.36 to 0.57)	0.22	0.61
	15 kPasc	1	0.10 +	(0.093 + 0.0753 * RV)	0.01	0.809	0.14 +	(− 0.17 to 0.61)	0.23	0.63
Shore durometer	NA	2	0.77*	(0.1559 + 0.0399 * RV)	0.59	0.016	0.71	(− 0.3 to 0.93)	0.06	0.17
STCM	5 mm	2	0.65 +	(− 0.0054 + 0.1872 * RV)	0.46	0.043	0.31 +	(− 0.99 to 0.82)	0.27	0.76
	10 mm	**2**	0.93***	(0.2128 + 0.0453 * RV)	**0.86**	** < 0.001**	0.8*	**(0.05 to 0.96)**	**0.05**	**0.13**
MyotonPRO Stiffness	**NA**	**2**	0.98***	(0.0193 + 0.0208 * RV)	**0.95**	** < 0.001**	0.99***	**(0.94 to 1)**	**0.00**	**0.01**
IndentoPRO	**2 mm**	**2**	0.97***	(0.0791 + 0.1756 * RV)	**0.94**	** < 0.001**	0.82*	**(− 0.03 to 0.96)**	**0.10**	**0.28**
	5 mm	**2**	0.97***	(0.0785 + 0.16 * RV)	**0.94**	** < 0.001**	0.81*	**(0.16 to 0.96)**	**0.09**	**0.25**
	**8 mm**	**2**	0.98***	(0.0887 + 0.1321 * RV)	**0.96**	** < 0.001**	0.75*	**(0.01 to 0.94)**	**0.07**	**0.20**
	**10 mm**	**2**	0.98***	(0.0714 + 0.121 * RV)	**0.96**	** < 0.001**	**0.71**	**(− 0.24 to 0.93)**	**0.08**	**0.22**
Ultrasound	**5 kPasc**	**2**	0.80**	(0.7334 + 0.8173 * RV)	**0.64**	**0.009**	**0.44**	**(− 1.63 to 0.87)**	**2.31**	**6.40**
	**10 kPasc**	**2**	0.89**	(0.6956 + 0.6704 * RV)	**0.79**	**0.001**	0.74*	**(− 0.17 to 0.94)**	**0.65**	**1.80**
	**15 kPasc**	**2**	0.95***	(0.7275 + 0.7006 * RV)	**0.90**	** < 0.001**	0.82*	**(0.1 to 0.96)**	**0.55**	**1.54**
Shore Durometer	**NA**	4	0.53	(0.026 + 0.0237 * RV)	0.28	0.144	0.51	(− 0.4 to 0.88)	0.04	0.10
STCM	5 mm	4	0.05 +	(0.0899 + 0.0554 * RV)	0.05	0.562	0.07 +	(− 0.7 to 0.69)	0.38	1.04
	10 mm	**4**	0.92***	(− 0.0502 + 0.0971 * RV)	**0.85**	** < 0.001**	0.93***	**(0.71 to 0.98)**	**0.03**	**0.09**
MyotonPRO Stiffness	**NA**	**4**	0.91***	(0.0076 + 0.0181 * RV)	**0.84**	** < 0.001**	0.94**	**(0.61 to 0.99)**	**0.01**	**0.01**
IndentoPRO	**2 mm**	**4**	0.96***	(− 0.0242 + 0.1158 * RV)	**0.92**	** < 0.001**	0.96***	**(0.82 to 0.99)**	**0.02**	**0.06**
	5 mm	**4**	0.96***	(− 0.0361 + 0.0996 * RV)	**0.92**	** < 0.001**	0.97**	**(0.38 to 0.99)**	**0.02**	**0.06**
	**8 mm**	**4**	0.92***	(0.0087 + 0.1037 * RV)	**0.85**	** < 0.001**	0.96**	**(0.67 to 0.99)**	**0.02**	**0.06**
	**10 mm**	**4**	0.97***	(− 0.0052 + 0.1471 * RV)	**0.93**	** < 0.001**	0.96***	**(0.71 to 0.99)**	**0.04**	**0.10**
Ultrasound	**5 kPasc**	**4**	0.92***	(− 0.2829 + 0.471 * RV)	**0.84**	** < 0.001**	0.82*	**(0.01 to 0.96)**	**0.27**	**0.75**
	**10 kPasc**	**4**	0.87**	(− 0.198 + 0.5139 * RV)	**0.76**	**0.0022**	0.87**	**(0.45 to 0.97)**	**0.26**	**0.71**
	**15 kPasc**	**4**	0.96***	(− 0.0585 + 0.5778 * RV)	**0.91**	** < 0.001**	0.94***	**(0.74 to 0.99)**	**0.16**	**0.45**

ICC estimates and their 95% CI were calculated using the R package “irr” version 0.84.1 based on a 2-way random-effects model with absolute agreement. + Not normally distributed data were log 10 transformed for ICC calculations; correlations were accordingly calculated with the Spearman’s rank correlation coefficient instead of the Pearson product-moment correlation. Values that show at least a high correlation (r > 0.8) are printed in bold type. NA. Data could not be measured. Layer 1: Cutis. Layer 2: Subcutaneous connective tissue. Layer 3: Fascia profunda (data not shown, since none of our assessment methods was able to yield significant stiffness differences between the different layer variants in this 1 mm thin layer). Layer 4: Erector spinae muscle.

Significant at the level *< 0.05; **< 0.01; ***< 0.001.

## Discussion

To our knowledge, this is the first study examining the reliability of the Shore Durometer, STCM, IndentoPRO, MyotonPRO, and ultrasound with force transducer on a MPTM mimicking different tissue layers of the human low-back region. The Shore Durometer, STCM, IndentoPRO and MyotonPRO reliably detected stiffness changes in three of the four MPTM layers. With the ultrasound method, only stiffness changes in layers thicker than 3 mm (i.e., SCT and ERS) could be measured reliably. No method could detect stiffness changes in the thin (1 mm) layer simulating the FPR.

The indentation and myotonometry devices included in this study are easy-to-use, with analog or digital displays allowing for an immediate reading of the stiffness-related measurement results. In addition, they are comparatively low in price^14,16,39^. As none of the tools was able to detect stiffness alterations in the very thin fascia profunda layer, we assume that a certain thickness greater than 1 mm of a layer must be present for the devices, particularly for indentation and myotonometry technologies, to detect changes. While the STCM and IndentoPRO can specifically be set to different indentation depths, those tools alone cannot provide a final information about which tissue layers may present with stiffness alterations. To draw this conclusion, the specific thickness of each layer must be known. Compared to indentation and myotonometry technologies, ultrasound imaging allows for the distinction of different tissue layers, particularly regarding tissue thickness^39^. Stiffness, however, can only be determined by ultrasound imaging by means of a force–deformation relationship. To quantify this ratio, the forces applied with the ultrasound transducer must be measured. The ultrasound set-up used in this study included a force gauge that was attached to a transducer by a firm, tight Velcro fastener. As this method could be sensitive to the alignment of the transducer and the force gauge, the used mounting might have impacted measurements as well as the resulting limits in measurement range observed in this study. Future studies should therefore use a firmer fixture when applying the same measurement set-up. Alternative, more cost-intensive technologies allowing for the evaluation of thickness as well as stiffness parameters comprise ultrasound elastography^40,41^ and magnetic resonance elastography^42,43^.

Previous studies have indicated that the biomechanical properties of layered tissue structures make it difficult to reliably determine tissue hardness. As one exemplary assessment technology, earlier work has used Shore Durometers to measure tissue stiffness in distinct tissue layers of the body, particularly so regarding the skin layer^26,33,44–48^. Recent research suggests that shore hardness, as measured by the Shore Durometer in the present study, is more representative of bulk tissue (i.e., skin plus underlying subcutaneous tissue) mechanics than it is of skin biomechanics alone. Stiffness may be influenced by the thickness of the individual tissue layers as well as by the size of the material probe. As a consequence, the thickness and size of individual tissue probes need to be known to draw conclusions about the stiffness values of the individual tissue layers^49,50^. While this prerequisite was met by the structure of the MPTM (individual tissue layers of homogenous materials with known thickness and stiffness properties), this might prove difficult in clinical practice. Ideally, thickness measurements with technologies such as ultrasound should be performed to secure these tissue properties. Alternatively, recent technologies such as ultrasound elastography or magnetic resonance elastography^42^ may serve as suitable methods to meet those requirements and to simultaneously determine soft tissue stiffness for all soft tissue layers including the fascia profunda^14,19,51,52^. While our reasoning relates to the thoracolumbar region, the findings presented here are transferable to other anatomical areas that have similar layered structures as the MPTM as well.

When relating our findings to the clinical practice, several considerations must be made. To select therapeutic interventions for low back patients, knowledge of the morphometry (e.g. thickness) and biomechanical characteristics (e.g. stiffness) of the thoracolumbar soft tissues in a healthy state as well as in the presence of low back pathology can serve as valuable decision criterion. In clinical practice, thickness of different soft tissue layers is primarily determined with ultrasound imaging^53^. With regards to stiffness assessment, manual examination has long been part of the clinical decision making. However, the validity and reliability of palpatory assessment have been described as poor^54–57^. SMT can be used by the clinical practitioner to document baseline measures and to track improvements over time and with interventions. The practitioner is posed with the challenge of finding evaluation methods able to discern stiffness changes across the different soft tissue layers as well as across the spectrum of different clinical presentations and populations. Our results support practitioners to choose appropriate measurement tools for different measurements depths.

In clinical practice, these findings should be linked with knowledge about morphological and biomechanical changes of the respective tissue layers in vivo in low back pain conditions. Various studies have examined these parameters: The cutis layer consists of the epidermis and dermis, with the latter providing most of the mechanical strength of this layer^58^. Changes in cutis thickness (measured by ultrasound) and hardness (assessed with Shore durometer) have been reported in relation to different static spinal postures^45^. Skin in the trunk dorsum was reported to become thicker (~ 17%) and softer (~ 39%) during spinal extension and to become thinner (~ 19%) and harder (~ 106%) during spinal flexion compared to a neutral prone position. Such changes are of note when it comes to the postural positioning of the patient during clinical evaluation. Furthermore these findings are of clinical significance as mechanoreceptors may respond differently due to their altered positioning and spacing, which could contribute to the decreased tactile acuity reported in chronic low back patients^59,60^.

The subcutaneous connective tissue (also termed hypodermis) consists of loose connective tissue and has been described to have mucous-like properties^61^. For the subcutaneous connective tissue thickness, an ultrasound study by Langevin and colleagues^4^ showed no significant differences between low back pain patients and asymptomatic controls. However, a recent retrospective study reviewing magnetic resonance images of low back patients revealed that the subcutaneous fat tissue thickness at the L1–L2 level proved to be superior to body mass index in predicting low back pain. The authors determined cutoff values to predict low back pain and spine degeneration for females (subcutaneous fat thickness > 8.45 mm) and males (> 9.4 mm)^62^. Furthermore, diet—particularly dietary salt intake—may influence lumbar subcutaneous edema, which could in turn influence non-specific low back pain^63^. Clinical practitioners should factor in such changes in subcutaneous tissue thickness and composition when examining thoracolumbar soft tissue structures.

For the fascia profunda layer, an ultrasound study found that the thoracolumbar fascia in people with chronic low back pain presented with 25% greater thickness compared to matched controls after adjusting for Body Mass Index^4^. Another biomechanical property of note for the clinical practice relates to the mobility between fascial tissue layers. Ultrasound investigations using cross-correlation analysis showed that the thoracolumbar shear strain was about 20% lower^3^ and deformability 28% smaller in patients with chronic low back pain during passive trunk flexion compared to controls^3^. While these findings may support the clinical practitioner in their evaluation, the contribution of the thoracolumbar fascia to low back pain remains less studied than their muscular counterparts^53^ and should be further examined in future investigations.

Regarding the role of the lumbar muscle layer in low back pain, the multifidus muscle has been extensively investigated^53^. Differences in cross-sectional area (males present with larger cross-sectional area than females), and asymmetry between sides (muscle atrophy has been described ipsilateral to the reported side of unilateral pain) have been reported^64^. Furthermore, fat infiltration of the multifidus muscle is common in adult low back patients, and especially among females. Interestingly, this finding seemed to be independent of overall body fat estimated through Body Mass Index^65^. This local change in tissue composition could alter biomechanical properties and consequently stiffness measurements and may therefore inform the practitioner’s assessment of the low back area. While these findings may inform the practitioner’s evaluation of the thoracolumbar region, standardized protocols and a broad range of reference values for thickness and stiffness of the different tissue layers, particularly as they relate to low back pain, are lacking. Reference databases for each of the soft tissue layer properties with regards to factors such as age, gender, body composition, ethnicity, and life-style differences are needed. In addition, it remains unclear how the relationship between different tissue layers changes in response to therapeutic intervention.

Even though our findings support the reliability and easy usability of the examined devices, several limitations of the present study need to be addressed. Previous research has recommended to use material phantoms to assess reliability of stiffness measurement devices^16^. Our gel pad model proved to be appropriate for compression stiffness measurements, characterized by a force applied perpendicular to the material. However, the buildup of the model did not allow for the consideration of shear strain, which would include force exerted sideways on the medium. In diagnostic measurements as well as in therapeutic applications, shear mobility between tissue layers may play an essential role when it comes to low-back health^35,66^. Accordingly, further development of the MPTM, allowing for shear mobility to be considered, would be desirable to produce valuable reliability and validity data for researchers and practitioners^67^.

For each MPTM layer, ten gel pads with varying stiffness parameters were manufactured. The stiffness alterations for those ten gel pads were determined individually for each layer. Accordingly, the absolute as well as relative stiffness changes for each layer set were not identical. While the ecological validity of the MPTM benefited from this approach, creating a MPTM with the same gradations for each layer would have increased the comparability of all measurements across the different tissue layers.

Furthermore, the thickness and stiffness values for the MPTM were determined from a literature search. To our knowledge, no current data map the various soft tissue layers of the thoracolumbar region by different ethnicities, age groups, or sexes. Therefore, none of these subgroups could be taken into account when we designed the MPTM. In reality, the layered structure of the low back may differ significantly for these subgroups. For instance, body composition has been reported to vary between different ethnicities^68^, and race-related errors in models of body composition assessment have been noted^69^. Such differences and assumption errors may result in varying biomechanical properties of the different tissue layers, as can easily be imagined for the subcutaneous connective tissue. Neighboring fields such as nutrition and cardiology are already considering differences in body compositions for their respective scopes^70,71^. Future research should be dedicated to establishing tissue layer properties for different subgroups, as such data may positively increase the validity of measurement devices and phantom models alike. Future work should furthermore refine existing measurement protocols^15^ for the examined SMT with defined measurement locations and reference values for different body regions in vivo.

## Data Availability

The datasets that will be used and/or analyzed during the current study will be available from the corresponding author on reasonable request.
